# A Clinical Case of Probable Sitosterolemia

**DOI:** 10.3390/ijms25031535

**Published:** 2024-01-26

**Authors:** Michishige Terasaki, Mikiko Izumi, Sho-ichi Yamagishi

**Affiliations:** 1 Division of Diabetes, Metabolism and Endocrinology, Showa University Graduate School of Medicine, 1-5-8 Shinagawa, Tokyo 142-8666, Japan; shoichi@med.showa-u.ac.jp; 2 Center for Clinical Genetics, Showa University Hospital, 1-5-8 Shinagawa, Tokyo 142-8666, Japan; mizumi@cnt.showa-u.ac.jp

**Keywords:** sitosterolemia, Met429Val, ABCG8, ezetimibe

## Abstract

Sitosterolemia is a rare genetic lipid disorder characterized by elevated plant sterols in the serum. A 24-year-old Japanese woman was referred to our hospital due to a high serum low-density lipoprotein cholesterol (LDL-C) level of 332 mg/dL. At first, she was suspected to suffer from familial hypercholesterolemia, and thus received lipid-lowering agents. Although her LDL-C level remained high (220 mg/dL) with diet therapy plus 10 mg/day rosuvastatin, it was drastically decreased to 46 mg/dL with the addition of 10 mg/day ezetimibe. Finally, her LDL-C level was well-controlled at about 70 mg/dL with 10 mg/day ezetimibe alone. Furthermore, while her serum sitosterol level was elevated at 10.5 μg/mL during the first visit to our hospital, it decreased to 3.6 μg/mL with the 10 mg/day ezetimibe treatment alone. These observations suggest that she might probably suffer from sitosterolemia. Therefore, targeted gene sequencing analysis was performed using custom panels focusing on the exome regions of 21 lipid-associated genes, including *ABCG5*, *ABCG8*, and familial hypercholesterolemia-causing genes (*LDL receptor*, *LDLRAP1*, *PCSK9*, and *apolipoprotein B*). We finally identified a heterozygous *ABCG8* variant (NM_022437.2:c.1285A>G or NP_071882.1:p.Met429Val) in our patient. The same gene mutation was detected in her mother. We report here a rare case exhibiting probable sitosterolemia caused by a heterozygous Met429Val variant in the *ABCG8* gene and additional unknown variants.

## 1. Introduction

Sitosterolemia (OMIM #210250, and #618666) is an extremely rare genetic lipid disorder characterized by elevated levels of plant sterols, including sitosterol [1,2]. Since patients with sitosterolemia exhibit elevated serum low-density lipoprotein cholesterol (LDL-C) levels with tendinous and tuberous xanthomas [2,3], their clinical manifestations resemble those with familial hypercholesterolemia (FH). Since the degree of LDL-C elevation and xanthomas appear to be more variable in patients with sitosterolemia than in those with FH [2], a certain proportion of patients with sitosterolemia may be under-reported or misdiagnosed as FH [1,3,4].

Sitosterolemia is caused by homozygous or double heterozygous mutations of the genes encoding ATP-binding cassette subfamily G member 5 (*ABCG5*) and member 8 (*ABCG8*) [2,5,6]. ABCG5 and ABCG8 proteins form heterodimers and act as a complex, which could play a role in the excretion of plant sterols and cholesterol into the bile and gut lumen [7]. Although the prevalence of these mutations is estimated to be 1 in 360,000 for *ABCG8* and 1 in 2.6 million for *ABCG5* [8], most Japanese patients with sitosterolemia are assumed to have *ABCG5* mutations [9].

We report here a rare Japanese patient exhibiting probable sitosterolemia caused by a heterozygous variant in *ABCG8* and additional unknown variants. Although her hypercholesterolemia remained high with 10 mg/day rosuvastatin, an inhibitor of hydroxymethylglutaryl-CoA (HMG-CoA) reductase plus diet therapy and additional therapy with ezetimibe drastically reduced her serum LDL-C level. Finally, her LDL-C level was well-controlled by the ezetimibe treatment alone without rosuvastatin. We performed a literature review to discuss the prevalence of heterozygous *ABCG8* gene mutations in patients with sitosterolemia.

## 2. Case Presentation

**Figure 1:**
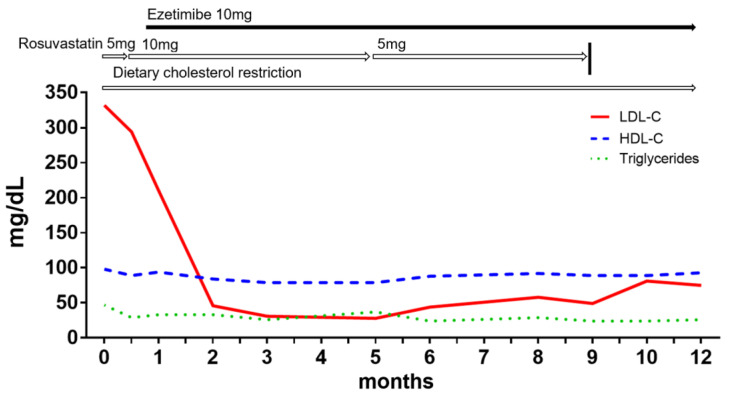
Time course of LDL-C, HDL-C, and triglyceride levels. LDL-C, low-density lipoprotein cholesterol; HDL-C, high-density lipoprotein cholesterol.

A 24-year-old Japanese woman was referred to our university hospital because her serum LDL-C level had been elevated to 332 mg/dL in a routine medical checkup. Her vital signs on the day of consultation were as follows: blood pressure, 90/57 mmHg; pulse rate, 60 beats per minute (regular); and temperature, 35.9 °C. Her body weight, height, and body mass index (BMI) were 46 kg, 164 cm, and 17.1 kg/m^2^, respectively. The results of the laboratory data are listed in Table 1. The patient did not present cutaneous or tuberous xanthomas. However, the intima–media complex thickness at the bifurcation of her internal carotid artery was 1.2 mm, and her Achilles tendons were thickened to 8.4/8.5 mm (assessed by radiography). At first, on the basis of these findings, she was suspected to have FH, and thus we recommended behavioral changes, including a calorie-restricted diet (1700 kcal/day) according to the dietary recommendations of the National Cholesterol Education Program/American Heart Association (NCEP/AHA) (Step 1: diet and regular exercise). At the same time, she started to receive 5 mg/day rosuvastatin for her hypercholesterolemia. Since the hypercholesterolemia was not controlled by the treatment, the dose of rosuvastatin was later increased to 10 mg/day. However, her serum LDL-C level still remained high at 220 mg/dL. Therefore, 10 mg/day ezetimibe therapy was added. As a result, her serum LDL-C level was drastically decreased to 46 mg/dL. Finally, 10 mg/day ezetimibe alone without rosuvastatin was found to be enough to control her LDL-C level at around 70 mg/dL (Figure 1).

The significant beneficial effect of ezetimibe, but not rosuvastatin, in our patient made us consider the possibility that she might suffer from sitosterolemia. Therefore, we evaluated her serum plant sterol levels. The sitosterol and campesterol levels at baseline were significantly elevated at 10.5 μg/mL and 21.7 μg/mL, respectively, both of which were normalized to 3.6 μg/mL and 5.2 μg/mL by the treatment with 10 mg/day ezetimibe alone (Table 2).

**Figure 2:**
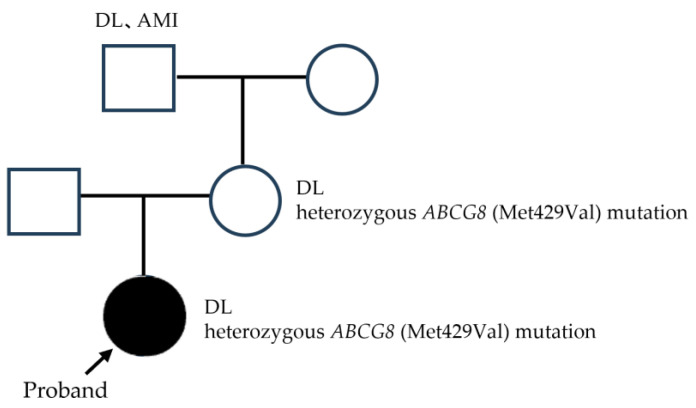
Pedigree analysis of the patient. A targeted analysis of genes was performed using a custom panel focusing on exome regions of 21 lipid-associated genes. The patient has a heterozygous Met429Val variant in the *ABCG8* gene. The same mutation was detected in her mother.

Then, we performed targeted gene sequencing with custom panels focusing on the exome regions of 21 lipid-associated genes. We finally identified a heterozygous *ABCG8* variant (NM_022437.2:c.1285A>G or NP_071882.1:p.Met429Val) in our patient, but no mutations were observed in *LDL receptor*, *LDLRAP1*, *PCSK9*, *apolipoprotein B*, or *ABCG5*. In addition, based on the genetic analysis, the same mutation in the *ABCG8* gene was also detected in the patient’s mother (Figure 2). The mother’s intima–media complex thickness at the bifurcation of the internal carotid artery was 1.4 mm, and her Achilles tendons were thickened to 8.0/8.1 mm (detected by X-ray). However, the mother’s serum LDL-C level was slightly elevated at 161 mg/dL, which was normalized to around 75 mg/dL using 10 mg/day ezetimibe alone.

## 3. Discussion 

Due to increased LDL-C level and Achilles tendon thickness, we first suspected that she may suffer from FH [10]. However, in the present study, the ezetimibe treatment alone without rosuvastatin normalized the patient's serum LDL-C level to about 70 mg/dL. The significant beneficial effect of ezetimibe alone, but not rosuvastatin, led us to speculate that there is a small possibility that she suffers from FH. Since our patient’s serum sitosterol and campesterol levels were elevated at baseline and drastically improved with the ezetimibe treatment alone, we suspected that she might suffer from sitosterolemia caused by loss-of-function mutations in *ABCG5* and/or *ABCG8*, both of which play a crucial role in the excretion of plant sterols from the intestine and liver [1,7]. Consequently, we identified a heterozygous variant in *ABCG8* (Met429Val), but not *ABCG5*, in our patient using targeted gene sequencing panels focusing on the exome regions of 21 lipid-associated genes. In the Japanese Guidelines for the Diagnosis and Treatment of Adult Familial Hypercholesterolemia 2022 [10], the Achilles tendon is judged to be thickened when its maximum diameter is 7.5 mm or greater for women. Therefore, although the patient does not have a formal diagnosis of sitosterolemia, according to these diagnostic criteria for sitosterolemia [11], our patient's diagnosis was probable sitosterolemia on the basis of the following findings; (A) clinical manifestation of tendon xanthomas, (B) high sitosterol level in serum ≥ 10 μg/mL, (C) exclusion of FH by targeted analysis of FH-causing genes, and (D) a heterozygous variant in the *ABCG8* gene.

To date, approximately 200 cases of sitosterolemia have been reported globally [12], most of which are caused by homozygous or double heterozygous mutations of the *ABCG5* and *ABCG8* genes [2,5,6]. The details of the nucleotide variant and its corresponding Met429Val mutation in the *ABCG8* gene are shown in Table 3. In the Japanese population database HGVD, the frequency of the Met429Val variant in the *ABCG8* gene is extremely low. We summarize the case studies of sitosterolemia with the Met429Val variant in the *ABCG8* gene in Japan in Table 4. Three patients with sitosterolemia that were homozygous for the Met429Val variant in the *ABCG8* gene showed high serum LDL-C and sitosterol levels of 374~823 mg/dL and 15~40 μg/mL, respectively [13]. An individual with double heterozygous mutations in *ABCG8* (Met429Val) and *ABCG5* also exhibited high serum LDL-C and sitosterol levels of 453 mg/dl and 15.9 μg/mL, respectively [14]. As far as we know, this is the first case of probable sitosterolemia with a heterozygous Met429Val mutation in *ABCG8* without a known *ABCG5* mutation, whose serum LDL-C and sitosterol levels were significantly elevated at 332 mg/dL and 10.5 μg/mL, respectively (Table 4). In a Japanese cohort study of primary hypercholesterolemic patients, carriers of the Met429Val mutant allele in the *ABCG8* gene were reported to have a slightly increased serum sitosterol level of 3.64 ± 1.26 μg/mL [15]. Therefore, the findings suggest that potential additional unknown genetic mutations or pathogenic variants other than *ABCG8* could be associated with sitosterolemia in our patient, which may partly explain why the patient’s mother did not have early development of coronary artery disease (CAD) or the same lipid profile, especially the markedly elevated LDL-C, as her daughter.

Recent clinical studies have shown that serum sitosterol levels could be a biomarker of sensitivity to ezetimibe therapy [16] and/or a surrogate marker of intestinal cholesterol absorption [17]. However, the serum sitosterol level is only weakly associated with dietary plant sterol intake and differs among individuals taking almost the same amounts of dietary sitosterol [18]. These observations suggest that interindividual differences in sterol metabolism, which were partly ascribed to the differences in the ability to excrete plant sterols into the bile by ABCG5 or ABCG8 rather than its intake, may affect the serum level of plant sterols. This is a reason why both sitosterolemia and elevated LDL-C levels were drastically improved by ezetimibe treatment alone in our patient. 

While dietary fiber and plant sterols are favorable for decreasing intestinal absorption of cholesterol in FH patients, the dietary intake of plant sterols and shellfish sterols, such as those in vegetable oils, nuts, avocados, and shellfish, should be avoided for the patients with sitosterolemia and variants in the *ABCG8* and/or *ABCG5* genes [19]. Moreover, statin is the first-line therapy for FH, whereas 10 mg/day ezetimibe is the best one for sitosterolemia. Heterozygous FH is the most common inherited disorder that causes high LDL-C levels in the serum; the prevalence of FH is estimated to be around 1 in 250 individuals in Japan [10]. Since the clinical manifestations of probable sitosterolemia caused by an *ABCG8* gene mutation observed here resemble the phenotype of FH, it is quite important to distinguish sitosterolemia from heterozygous FH among hypercholesterolemic patients with tendon xanthomas by measuring serum plant sterol levels and/or performing a genetic analysis of *ABCG8* and *ABCG5* if the patient’s high LDL-C level is drastically improved by the co- or mono-treatment with ezetimibe.

**Figure 3:**
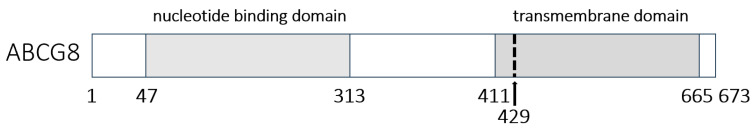
A mutation in the *ABCG8* gene. ABCG8 contains nucleotide-binding and transmembrane domains. The nucleotide-binding domain is composed of amino acids 47 to 313. The transmembrane domain is composed of amino acids 411 to 665. Met429Val mutation in *ABCG8* is located in the transmembrane domain.

**Figure 4:**
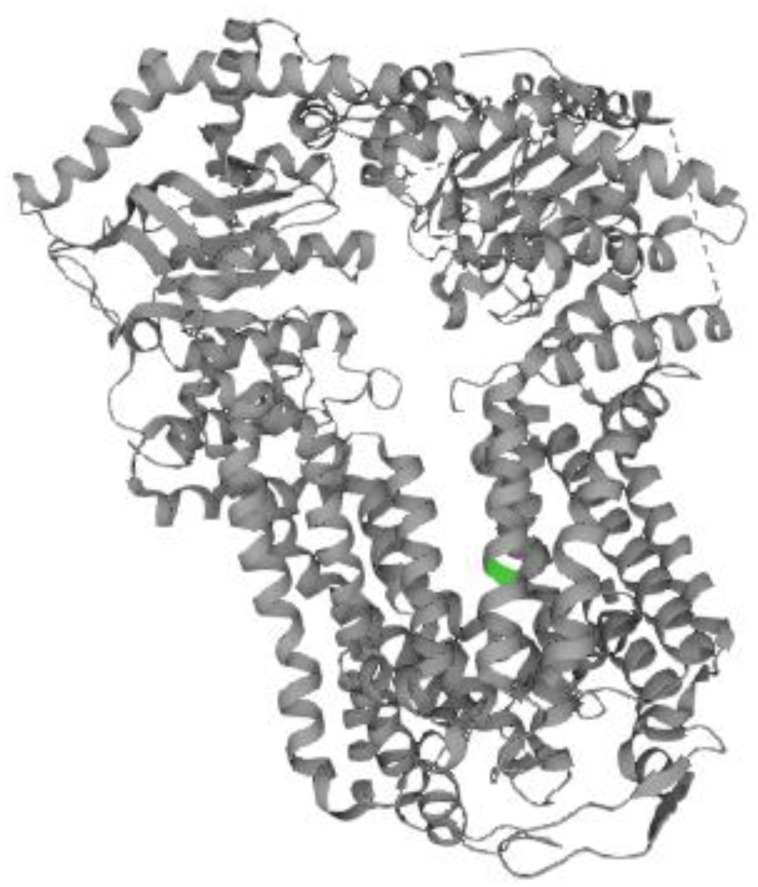
Crystal structure of *ABCG8*. *ABCG8* Met429Val mutation is shown in green.

Genome-wide association studies (GWASs) have shown remarkable associations between *ABCG5* and *ABCG8* gene variations and serum LDL-C levels and the prevalence of CAD [20,21]. Indeed, patients with sitosterolemia with homozygous variants in the *ABCG8* or *ABCG5* gene have been reported to have CAD and exhibit increased serum LDL-C as well as sitosterol levels [22]. Furthermore, an elevation of serum sitosterol and LDL-C levels has been observed in heterozygous carriers of *ABCG5* mutations [23]. In addition, there was a recent case report of a 6-month-old baby that had sitosterolemia with a high LDL-C level and xanthomas caused by heterozygous nonsense and two missense mutations in the *ABCG8* gene [24]. From the clinical data of our patient, heterozygous mutations in the *ABCG8* gene might also be associated with both increased serum sitosterol and LDL-C levels. Because the Met429Val variant in *ABCG8* is predicted to be located in the first transmembrane domain at the N-terminal (Figure 3 and Figure 4), the variant may be a causative factor for high serum sitosterol and LDL-C levels.

The present study has some potential limitations. First, since whole-genome sequencing was not performed, we cannot completely rule out mutations other than ones in the *ABCG8* gene in our case. Since her mother who is also the carrier of this mutation did not exhibit markedly elevated LDL-C levels, apart from the heterozygous Met429Val mutation in *ABCG8*, it is highly probable that additional unknown variants that could not be identified by our targeted gene sequencing panels may also contribute to the sitosterolemia in our patient. In addition, sequencing of the promoter regions of the *ABCG8* and *ABCG5* genes was not conducted. Some pathogenic nucleotide substitution in the promoter regions of *ABCG8* and *ABCG5* gene may modify our patient’s sitosterol and LDL-C levels. Therefore, it would be interesting to adopt an animal model with a heterozygous Met429Val mutation in *ABCG8* to study the pathological role of this mutation in sitosterolemia. Second, whether the heterozygous Met429Val mutation in *ABCG8* alone could promote atherosclerotic CAD remains unknown. Third, the serum sitosterol and campesterol levels were not measured in the patient’s mother. Therefore, we do not know how much the heterozygous Met429Val mutation in *ABCG8* alone contributed to the increased serum sitosterol level in our patient. Fourth, there still exists the possibility that our patient may have FH as the level of sitosterol was only two-fold higher and was at the lower boundary of the diagnostic criteria for sitosterolemia [11,22,25]. Indeed, the prevalence of *ABCG5*/*ABCG8* heterozygous carriers has been reported to be 8.3 times higher among patients with FH compared to controls [11,22,25]. Furthermore, heterozygous carriers of a loss of function variant in *ABCG5* had significantly increased sitosterol and LDL-C levels with a 2-fold higher risk of CAD [23].

In conclusions, we report a rare case exhibiting probable sitosterolemia caused by a heterozygous Met429Val variant in the *ABCG8* gene and additional unknown variants, which was associated with increased serum sitosterol and LDL-C levels. 

## 4. Materials and Methods

### 4.1. Measurement of Serum Lipids

The serum LDL-C, HDL-C, and triglyceride levels were measured by conventional direct methods as previously described [26]. Serum sitosterol and campesterol levels were measured using the liquid chromatography with tandem mass spectrometry (LC-MS/MS) method (BML, Inc., Tokyo, Japan).

### 4.2. Targeted Gene Sequencing Analysis

The targeted gene sequencing analysis was performed using custom panels focusing on the exome regions of 21 lipid-associated genes, including familial hypercholesterolemia-causing genes (*LDL receptor*, *LDLRAP1*, *PCSK9*, *apolipoprotein B*), *ABCG5*, and *ABCG8* as described previously [14]. The generated Fastq format files were aligned with the human reference genome sequence using Genome Reference Consortium Human Build 38/Human Genome 38 (GRCh38/hg38).

### 4.3. Bioinformatics Analysis for Predicting Pathogenicity

A Genome Aggregation Database was obtained from gnomAD (https://gnomad.broadinstitute.org/, accessed on 30 November 2023). The Single-Nucleotide Polymorphism Database was from https://www.ncbi.nlm.nih.gov/snp/ (accessed on 30 November 2023). The Human Genetic Variation Database was from http://www.hgvd.genome.med.kyoto-u.ac.jp/ (accessed on 30 November 2023). The original and mutant ABCG8 structure images were taken from the Protein Data Bank (https://www.rcsb.org/, accessed on 30 November 2023).

## Figures and Tables

**Table 1:** Laboratory data of the patient at baseline.

Hematological data	
White blood cells (WBCs)	2600/µL
Hemoglobin (Hb)	12.7 g/dL
Hematocrit	38.3%
Platelets (PLTs)	17.5 × 10^4^/uL
Biochemical data	
Total protein (TP)	7.0 g/dL
Albumin (Alb)	4.5 g/dL
Total bilirubin (T-Bil)	1.0 mg/dL
Blood urea nitrogen (BUN)	9.1 mg/dL
Creatine (Cr)	0.65 mg/dL
Creatine kinase (CK)	118 IU/L
Aspartate transaminase (AST)	23 IU/L
Alanine transaminase (ALT)	19 IU/L
γ-glutamyl transpeptidase (γ-GTP)	8 IU/L
Triglycerides	47 mg/dL
High-density lipoprotein cholesterol (HDL-C)	98 mg/dL
Low-density lipoprotein cholesterol (LDL-C)	332 mg/dL
Apolipoprotein A-I (ApoA-I)	190 mg/dL
Apolipoprotein A-II (ApoA-II)	20.1 mg/dL
Apolipoprotein B (ApoB)	201 mg/dL
Apolipoprotein C-II (ApoC-II)	6.5 mg/dL
Apolipoprotein C-III (ApoC-III)	12.8 mg/dL
Apolipoprotein E (ApoE)	7.8 mg/dL
Glucose	71 mg/dL
Hemoglobin A1c (%)	5.4%
Thyroid-stimulating hormone (TSH)	2.02 µIU/L
Free triiodothyronine (FT3)	1.74 pg/mL
Free thyroxine (FT4)	0.73 ng/mL
Urinalysis	
Protein	−
Glucose	−
Ketone body	−

−: none.

**Table 2:** Serum plant sterol levels in the patient.

	At Baseline	After Ezetimibe Treatment Alone
Sitosterol	10.5 μg/mL	3.6 μg/mL
Campesterol	21.7 μg/mL	5.2 μg/mL

Reference intervals: sitosterol 1.03–4.45 μg/mL; campesterol 2.19–8.34 μg/mL in Japanese women.

**Table 3:** Details of the nucleotide variant and its corresponding amino acid in the *ABCG8* gene.

Nucleotide	Amino Acid	Genome Aggregation	dbSNP	HGVD
c.1285A>G	p.Met429Val	0.000342 (52/152032)	rs147194762	0.001292 (2/1560)

**Table 4:** Summary of patients with sitosterolemia exhibiting Met429Val variant in *ABCG8* gene in Japan.

No.	Age	Gender	Genetic Mutation	LDL-C (mg/dL)	Sitosterol (μg/mL)	Xanthomas	Reference
1	8-month	F	homozygous *ABCG8* mutation	832	36.5	Yes	[13]
2	3-month	F	homozygous *ABCG8* mutation	554	40	Yes	[13]
3	1-year	F	double heterozygous *ABCG8* and *ABCG5* mutations	453	15.9	Yes	[14]
4	47-year	M	homozygous *ABCG8* mutation	374	15	Yes	[13]
5	24-year	F	heterozygous *ABCG8* mutation	332	10.6	Yes	The present case

Reference intervals: sitosterol 1.03–4.45 μg/mL in women and 0.99–3.88 μg/mL in men in Japan.

## Data Availability

Data are contained within the article.

## References

[1] Berge K.E., Tian H., Graf G.A., Yu L., Grishin N.V., Schultz J., Kwiterovich P., Shan B., Barnes R., Hobbs H.H. (2000). Accumulation of Dietary Cholesterol in Sitosterolemia Caused by Mutations in Adjacent ABC Transporters. Science.

[2] Tada H., Nohara A., Inazu A., Sakuma N., Mabuchi H., Kawashiri M.A. (2018). Sitosterolemia, Hypercholesterolemia, and Coronary Artery Disease. J. Atheroscler. Thromb..

[3] Sakuma N., Tada H., Mabuchi H., Hibino T., Kasuga H. (2017). Lipoprotein Apheresis for Sitosterolemia. Ann. Intern. Med..

[4] Tada H., Okada H., Nomura A., Yashiro S., Nohara A., Ishigaki Y., Takamura M., Kawashiri M.A. (2019). Rare and Deleterious Mutations in ABCG5/ABCG8 Genes Contribute to Mimicking and Worsening of Familial Hypercholesterolemia Phenotype. Circ. J..

[5] Buonuomo P.S., Iughetti L., Pisciotta L., Rabacchi C., Papadia F., Bruzzi P., Tummolo A., Bartuli A., Cortese C., Bertolini S. (2017). Timely diagnosis of sitosterolemia by next generation sequencing in two children with severe hypercholesterolemia. Atherosclerosis.

[6] Bastida J.M., Girós M.L., Benito R., Janusz K., Hernández-Rivas J.M., González-Porras J.R. (2019). Sitosterolemia: Diagnosis, Metabolic and Hematological Abnormalities, Cardiovascular Disease and Management. Curr. Med. Chem..

[7] Graf G.A., Yu L., Li W.P., Gerard R., Tuma P.L., Cohen J.C., Hobbs H.H. (2003). ABCG5 and ABCG8 are obligate heterodimers for protein trafficking and biliary cholesterol excretion. J. Biol. Chem..

[8] Hooper A.J., Bell D.A., Hegele R.A., Burnett J.R. (2017). Clinical utility gene card for: Sitosterolaemia. Eur. J. Hum. Genet..

[9] Kidambi S., Patel S.B. (2008). Sitosterolaemia: Pathophysiology, clinical presentation and laboratory diagnosis. J. Clin. Pathol..

[10] Harada-Shiba M., Arai H., Ohmura H., Okazaki H., Sugiyama D., Tada H., Dobashi K., Matsuki K., Minamino T., Yamashita S. (2023). Guidelines for the Diagnosis and Treatment of Adult Familial Hypercholesterolemia 2022. J. Atheroscler. Thromb..

[11] Tada H., Nomura A., Ogura M., Ikewaki K., Ishigaki Y., Inagaki K., Tsukamoto K., Dobashi K., Nakamura K., Hori M. (2021). Diagnosis and Management of Sitosterolemia 2021. J. Atheroscler. Thromb..

[12] Xia Y., Duan Y., Zheng W., Liang L., Zhang H., Luo X., Gu X., Sun Y., Xiao B., Qiu W. (2022). Clinical, genetic profile and therapy evaluation of 55 children and 5 adults with sitosterolemia. J. Clin. Lipidol..

[13] Tada H., Kojima N., Yamagami K., Takamura M., Kawashiri M.-A. (2022). Clinical and genetic features of sitosterolemia in Japan. Clin. Chim. Acta.

[14] Tada H., Nomura A., Yamagishi M., Kawashiri M.-A. (2018). First case of sitosterolemia caused by double heterozygous mutations in ABCG5 and ABCG8 genes. J. Clin. Lipidol..

[15] Miwa K., Inazu A., Kobayashi J., Higashikata T., Nohara A., Kawashiri M., Katsuda S., Takata M., Koizumi J., Mabuchi H. (2005). ATP-binding cassette transporter G8 M429V polymorphism as a novel genetic marker of higher cholesterol absorption in hypercholesterolaemic Japanese subjects. Clin. Sci. (Lond.).

[16] Hagiwara N., Kawada-Watanabe E., Koyanagi R., Arashi H., Yamaguchi J., Nakao K., Tobaru T., Tanaka H., Oka T., Endoh Y. (2017). Low-density lipoprotein cholesterol targeting with pitavastatin + ezetimibe for patients with acute coronary syndrome and dyslipidaemia: The HIJ-PROPER study, a prospective, open-label, randomized trial. Eur. Heart J..

[17] Dayspring T.D., Varvel S.A., Ghaedi L., Thiselton D.L., Bruton J., McConnell J.P. (2015). Biomarkers of cholesterol homeostasis in a clinical laboratory database sample comprising 667,718 patients. J. Clin. Lipidol..

[18] Kempen H., de Knijff P., Boomsma D., van der Voort H., Leuven J.G., Havekes L. (1991). Plasma levels of lathosterol and phytosterols in relation to age, sex, anthropometric parameters, plasma lipids, and apolipoprotein E phenotype, in 160 Dutch families. Metabolism.

[19] Farzam K., Morgan R.T. (2023). Sitosterolemia.

[20] Willer C.J., Schmidt E.M., Sengupta S., Peloso G.M., Gustafsson S., Kanoni S., Ganna A., Chen J., Buchkovich M.L., Mora S. (2013). Discovery and refinement of loci associated with lipid levels. Nat. Genet..

[21] Lu X., Peloso G.M., Liu D.J., Wu Y., Zhang H., Zhou W., Li J., Tang C.S., Dorajoo R., Li H. (2017). Exome chip meta-analysis identifies novel loci and East Asian-specific coding variants that contribute to lipid levels and coronary artery disease. Nat. Genet..

[22] Tada M.T., Rocha V.Z., Lima I.R., Oliveira T.G.M., Chacra A.P., Miname M.H., Nunes V.S., Nakandakare E.R., Castelo M.H.C.G., Jannes C.E. (2022). Screening of *ABCG5* and *ABCG8* Genes for Sitosterolemia in a Familial Hypercholesterolemia Cascade Screening Program. Circ. Genom. Precis. Med..

[23] Nomura A., Emdin C.A., Won H.H., Peloso G.M., Natarajan P., Ardissino D., Danesh J., Schunkert H., Correa A., Bown M.J. (2020). Heterozygous ABCG5 Gene Deficiency and Risk of Coronary Artery Disease. Circ. Genom. Precis. Med..

[24] Hashimoto N., Dateki S., Suzuki E., Tsuchihashi T., Isobe A., Banno S., Kageyama T., Maeda N., Hatabu N., Sato R. (2020). Compound heterozygous variants in the ABCG8 gene in a Japanese girl with sitosterolemia. Hum. Genome Var..

[25] Miroshnikova V.V., Romanova O.V., Ivanova O.N., Fedyakov M.A., Panteleeva A.A., Barbitoff Y.A., Muzalevskaya M.V., Urazgildeeva S.A., Gurevich V.S., Urazov S.P. (2021). Identification of novel variants in the *LDLR* gene in Russian patients with familial hypercholesterolemia using targeted sequencing. Biomed. Rep..

[26] Terasaki M., Yashima H., Mori Y., Saito T., Matsui T., Hiromura M., Kushima H., Osaka N., Ohara M., Fukui T. (2020). A Dipeptidyl Peptidase-4 Inhibitor Inhibits Foam Cell Formation of Macrophages in Type 1 Diabetes via Suppression of CD36 and ACAT-1 Expression. Int. J. Mol. Sci..

